# Radiation‐induced morphea of the breast—A case series

**DOI:** 10.1002/ski2.148

**Published:** 2022-06-29

**Authors:** Paula Finnegan, Lisa Kiely, Catriona Gallagher, Sarah Ni Mhaolcatha, Linda Feeley, Jim Fitzgibbon, Jessica White, John Bourke, Lesley Ann Murphy

**Affiliations:** ^1^ Department of Dermatology University Hospital Limerick Dooradoyle Limerick Ireland; ^2^ Department of Dermatology South Infirmary Victoria University Hospital Cork Ireland; ^3^ Department of Histopathology Cork University Hospital Wilton, Cork Ireland

## Abstract

Radiation‐induced morphea (RIM) is a rare but recognized late complication of radiotherapy. It was first described in 1905, not long after the initial discovery of X‐rays by Roentgen. Characterized by the deposition of excess collagen in the dermis, it results in thickening of the skin. Its frequency is approximately 2 in 1000. We present a series of three cases involving patients receiving radiotherapy treatment for breast cancer, each of which subsequently developed RIM. Because of its rarity, RIM is often misdiagnosed as infection or metastatic disease. This can lead to delayed diagnosis and treatment, leading to poorer outcomes such as chronic pain issues. Early dermatological involvement and tissue sampling to examine histopathological features can avoid this, leading to better care and improved results. A variety of treatment options are available, ranging from topical to systemic, with early induction more likely to result in a positive response.

## Graphical abstract

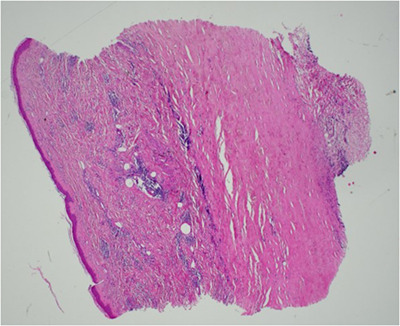

1

**What is already known about this topic?**

- Radiation‐induced morphea is a rare, poorly understood complication of radiotherapy that is often misdiagnosed
- Accurate diagnosis can only be reached by combining both clinical and histopathological features
- Early treatment induction is more likely to result in a positive response and avoid potential long‐term complications

**What does this study add?**

- The aim of this case series is to raise awareness of this rare complication in order to prevent misdiagnosis
- This case series highlights the importance of early involvement by dermatology allow for prompt recognition and expedite early induction of treatment

## 2 INTRODUCTION

Breast cancer is the commonest cancer to affect females. Radiotherapy is often used as part of its treatment as it has been shown to both reduce local recurrence and improve survival.^1^ However, radiotherapy can have multiple complications, including skin reactions in up to 90% of cases.^2^, ^3^ Radiation‐induced morphea (RIM) is a rare late skin complication of radiotherapy where excess collagen is deposited in the dermis, leading to thickening of the skin.^2^ It is also known as post‐irradiation morphea, radiation‐port morphea, radiation‐port scleroderma, radiation‐induced scleroderma, localized scleroderma and circumscribed scleroderma.^4^ First described in 1905, not long after the initial discovery of x‐rays by Roentgen, its frequency is approximately 2 in 1000.^5^ We describe three cases of patients receiving radiotherapy for breast cancer, each of which subsequently developed RIM. Because of its rarity, it is often misdiagnosed as infection, radiation‐induced fibrosis, or metastatic disease.^4^ This can lead to delayed diagnosis and treatment, resulting in poorer outcomes such as breast disfiguration and chronic pain.^1^, ^4^

## 3 CASE PRESENTATIONS

**FIGURE 1:**
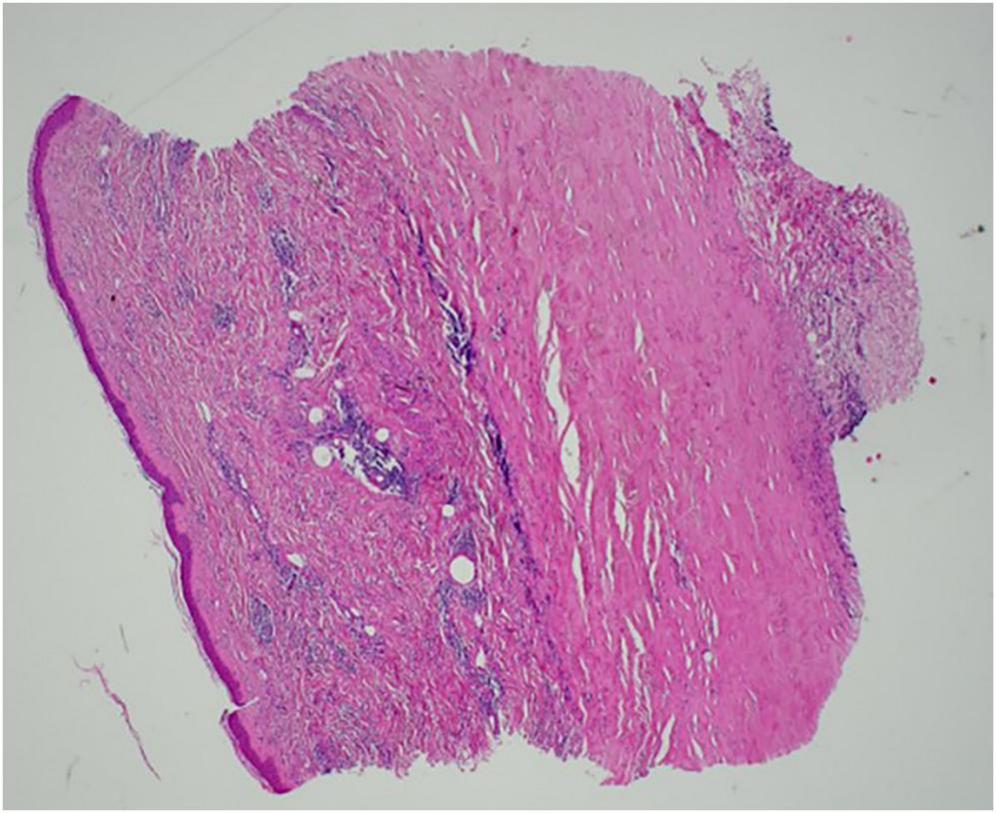
Case 1. Skin with prominent dermal sclerosis, loss of adnexal structures and superficial and deep perivascular lymphoplasmacytic inflammation

Case 1 A 77‐year‐old Caucasian female with a history of left‐sided breast cancer (grade II invasive ductal carcinoma) had been treated with wide local excision, sentinel lymph node biopsy, radiotherapy and chemotherapy in 2010. She reported non‐painful left breast erythema 1 year following completion of radiotherapy. Initially thought to be tumour reactivation, she underwent a punch biopsy – this showed dermal sclerosis with superficial and deep perivascular lymphocytic and plasmacytic infiltrate extending to fat, suggestive of RIM (see Figure 1). Few atypical fibroblasts were also seen. She was referred to dermatology who confirmed the diagnosis, and commenced a topical regime of mometasone furoate (Elocon) mane and Calcipotriol (Dovonex) nocte. Her skin colour gradually improved and the breast tissue texture in the affected area softened over 2 years. She regularly attends follow‐up.

**FIGURE 2:**
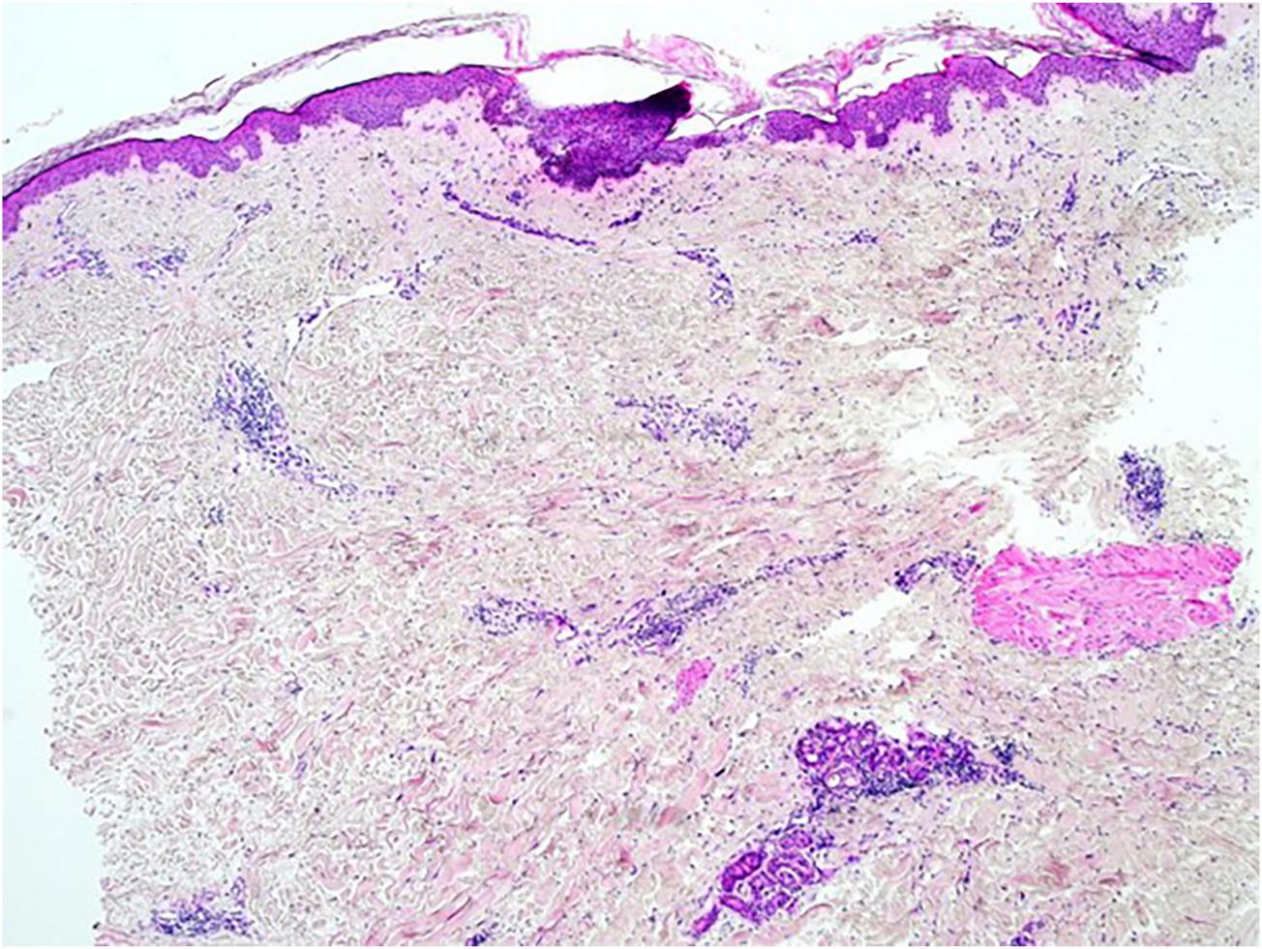
Case 2. Superficial dermis demonstrating perivascular mononuclear infiltrate. No evidence of sclerosis

Case 2 A 56‐year‐old Caucasian female with a history of right‐sided breast cancer (ductal carcinoma in situ) underwent a lumpectomy and radiotherapy in 2019. One year later, she was referred to dermatology with a persistent rash over the right breast, described as painful, heavy and itchy. Histopathology showed superficial and deep perivascular mononuclear infiltrate, arranged tightly around blood vessels, and extending into the subcutaneous fat and between collagen bundles in the dermis where there were occasional multinucleate giant cells (see Figure 2). There was no evidence of sclerosis. Radiation fibroblasts were present. The histopathological differential diagnosis included radiation‐induced dermatitis or early stage RIM. On clinical examination, a diagnosis of RIM was confirmed. She was commenced on a topical regime of Betamethasone/Calcipotriol (Enstilar foam) mane, and clobetasol propionate (Dermovate) ointment nocte. Her symptoms greatly improved within 8 weeks, and she is regularly followed‐up.

**FIGURE 3:**
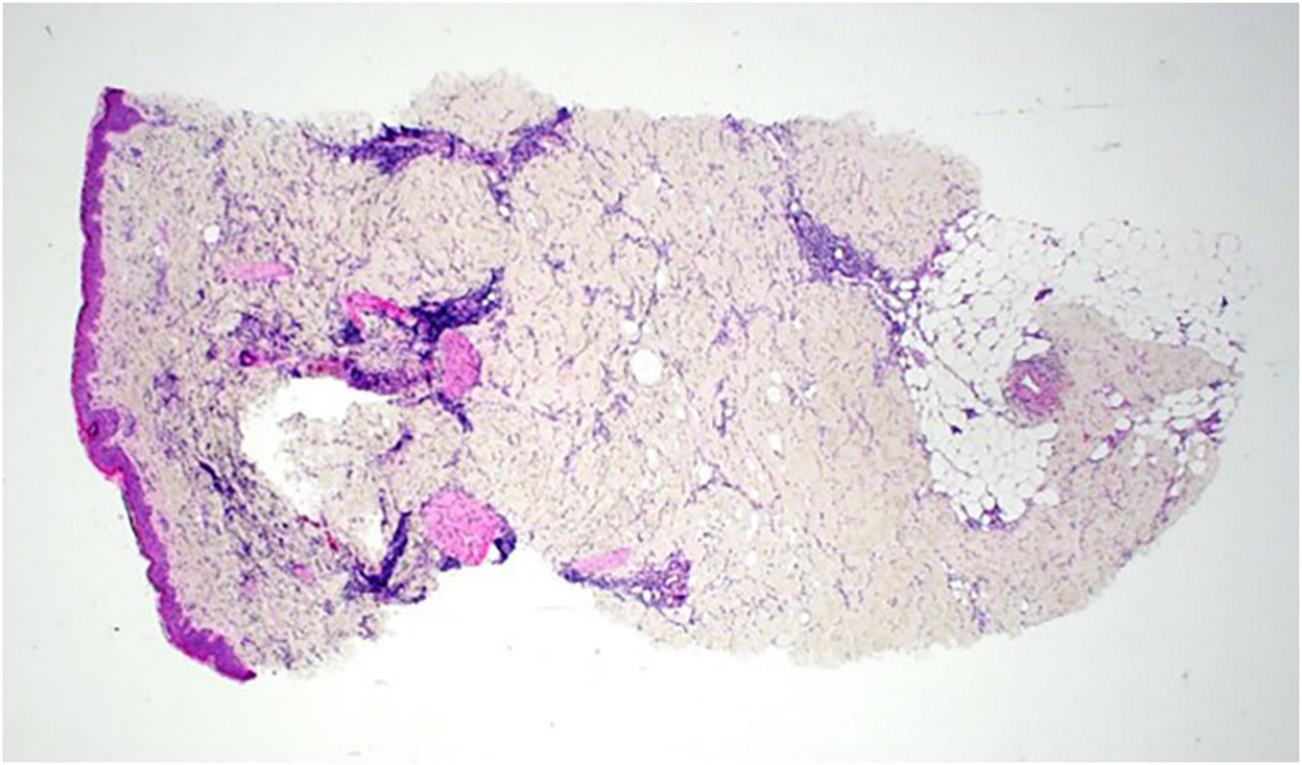
Case 3. Punch biopsy showed dermal fibrosis with a “squared‐off” profile and distinct delineation between fibrotic dermis and underlying subcutaneous tissue; patchy perivascular and interstitial chronic inflammation, including conspicuous plasma cells with unremarkable overlying epidermis

Case 3 A 62‐year‐old female with a history of left‐sided breast cancer (grade II invasive ductal carcinoma) had undergone treatment with a wide local excision and sentinel lymph node biopsy, followed by adjuvant radiotherapy in 2015. She was referred 5 years later with a 6‐month history of a well‐demarcated, erythematous pruritic rash in the superolateral and inframammary fold of the left treated breast. The skin subsequently became indurated on the lateral aspect of the breast. Punch biopsy showed dermal fibrosis with a “squared‐off” profile and distinct delineation between fibrotic dermis and underlying subcutaneous tissue (see Figure 3). There was patchy perivascular and interstitial chronic inflammation, including conspicuous plasma cells with unremarkable overlying epidermis. Atypical fibroblasts were also seen. Fungal profiles or significantly increased dermal mucin were not identified on special stains. Clinical and histopathological findings were in keeping with RIM. A trial of topical mometasone furoate (Elocon) and Calcipotriol (Dovonex) was commenced, and a clinical response is awaited.

## 4 DISCUSSION

With an approximate incidence of 0.2%, RIM is rare.^1^ The timing of its onset can vary from months to years post‐radiotherapy.^1^, ^4^, ^5^, ^6^ It is more common in women, and the breast is the most common location for it to occur.^3^ It has two phases—first, an initial inflammatory phase involving sudden onset of erythema and localized inflammation, mimicking cellulitis.^6^, ^7^ Histopathological findings here include perivascular and peri‐adnexal lymphocytic infiltrate and slight dermal collagen thickening.^6^ This is then followed by a later ‘burn‐out’ phase where there is reduced inflammation with increasing fibrosis and hyperpigmentation, resulting in fibrotic retraction, a *peau d'orange* appearance, and significant anatomical disfigurement.^1^, ^2^, ^3^, ^6^ Histopathology here shows prominent fibrosis with sclerotized collagen fibres and loss of the initial lymphocytic infiltrate.^6^

RIM is associated with breast cancer, larger breast size, and systemic sclerosis.^1^, ^5^ RIM is not related to age, radiation dose, patient age, severity of the initial acute reaction, or prognosis of the underlying malignancy.^1^, ^3^, ^7^ Disease course was found to be more severe in patients with a history of obesity, active smoking status, co‐existing auto‐immune disease, and breast implantation.^6^

RIM is often misdiagnosed as radiation‐induced fibrosis.^2^, ^5^ These two separate conditions can be distinguished by their timing, clinical features, and histopathological findings. RIM is more likely to be a late complication, with an abrupt onset involving erythema and induration.^5^ Its histopathological findings include dermal inflammatory infiltrates.^5^ RIM may also spread outside of the irradiated field, unlike radiation‐induced fibrosis which stays localized.^2^, ^6^ Other differential diagnoses for RIM include metastatic disease, cellulitis, fat necrosis, and radiation dermatitis. Biopsy is vital for differentiating between these.^2^, ^6^

The pathology leading to RIM is poorly understood. There are two theories currently. First, that the radiation stimulates the formation of neoantigens which trigger the release of transforming growth‐factor beta (TGF‐*β*).^2^, ^5^ TGF‐*β* leads to an increased level of fibrosis by activating fibroblasts and causing excess collagen production. The second theory postulates that fibroblasts exposed to radiation differentiate into more active forms, leading to an imbalance of fibroblast activity, resulting in excess collagen production and fibrosis.^5^

There are multiple treatment options available for RIM, ranging from topical to systemic and interventional.^7^, ^8^ The treatment approach is similar to that of idiopathic morphea.^3^, ^5^ These options include: antibiotics (oral or systemic), steroids (topical, intralesional or systemic), light therapy (Ultraviolet A), other topical agents (e.g. hyaluronidase, calcipotriol), other systemic agents, photodynamic therapy (PDT), and in rare cases, surgical intervention.^1^, ^5^, ^6^, ^8^, ^9^ Alternative steroid‐sparing agents used for managing RIM include: methotrexate, colchicine, azathioprine, cyclophosphamide, cyclosporin, tofacitinib, acitretin, mycophenolate or plasmaphaeresis.^1^, ^5^, ^6^ Psoralen‐and‐ultraviolet‐light‐A (PUVA) in particular has been found to have excellent results with reduced inflammation and improved skin texture.^1^, ^2^, ^7^ In cases where the patient is unable to commit to attending regular PUVA treatments, PDT may be considered as an alternative approach – Papanikolaou et al report such a case, with evidence of clinical improvement being demonstrated within 1 month of treatment.^8^ Regarding a surgical approach to management, due to the rarity of the condition there is a paucity of data available. Walsh et al reported one case of a patient undergoing a mastectomy for the symptomatic relief from RIM, however they were lost to follow‐up.^9^ Dancey et al. also reported successful surgical intervention for a case of RIM where modified breast reduction was performed—their patient reported resolution of symptoms and high satisfaction levels with no recurrence at follow‐up 6 months later.^10^ Regardless of treatment modality, early induction of treatment is more likely to result in a positive response,^1^ especially if started during the first phase of RIM.

## 5 CONCLUSION

The pathogenesis of RIM is poorly understood. An accurate diagnosis can only be reached by combining both clinical findings with histopathological features. Early dermatology involvement is vital to allow for prompt recognition and treatment induction, to ensure positive outcome and avoid potential long‐term sequelae such as chronic pain and anatomical distortion.

## AUTHOR CONTRIBUTIONS

**Paula Finnegan**: Conceptualization (equal); Data curation (lead); Writing – original draft (lead); Writing – review & editing (equal). **Lisa Kiely**: Data curation (supporting); Writing – review & editing (equal). **Catriona Gallagher**: Conceptualization (equal); Supervision (supporting). **Sarah Ni Mhaolcatha**: Writing – review & editing (supporting). **Linda Feeley**: Writing – review & editing (supporting). **Jim Fitzgibbon**: Writing – review & editing (supporting). **Jessica White**: Writing – review & editing (supporting). **John Bourke**: Supervision (supporting). **Lesley Ann Murphy**: Conceptualization (lead); Supervision (lead).

## CONFLICTS OF INTEREST

The author declares that there is no conflict of interest that could be perceived as prejudicing the impartiality of the research reported.

## ETHICS STATEMENT

The patients in this manuscript have given informed consent to publication.

## Data Availability

Data sharing not applicable – no new data generated.
